# Presentation of HIV‐Associated Thrombotic Microangiopathy and Response to Therapeutic Plasma Exchange: A 10‐year Retrospective Single‐Centre Cohort Study

**DOI:** 10.1002/hsr2.71932

**Published:** 2026-03-04

**Authors:** Malcolm Davies, Sheetal Chiba, Sayuri Harishun, Chandni Dayal, Zaheera Cassimjee

**Affiliations:** ^1^ University of the Witwatersrand Johannesburg South Africa; ^2^ Division of Nephrology Helen Joseph Hospital Johannesburg South Africa

**Keywords:** acute kidney injury, HIV, therapeutic plasma exchange, thrombotic microangiopathy

## Abstract

**Background:**

HIV is a significant aetiological factor in thrombotic microangiopathy (TMA) in regions of high seroprevalence, but description of HIV‐associated TMA (HIV‐TMA) remains limited to small case series. We sought to describe the presentation, complications of TPE, and mortality and renal outcomes of HIV‐TMA.

**Methods:**

We retrospectively reviewed 98 cases of HIV‐TMA treated with therapeutic plasma exchange (TPE) between 1/1/2010 and 31/12/2020. The effect of HIV infection and clinical presentation on mortality, TPE complications, and renal outcomes were analysed using regression analysis.

**Results:**

TMA is associated with advanced HIV infection (median CD4 count 151 × 106/mm^3^ cells and median viral load 117500 copies/mL), usually occurs in the absence of antiretroviral therapy (ART), and shows a predilection for young Black women, reflecting TMA risk factors and local demographics of the HIV pandemic. Neurological deficit is the most common presenting feature (54.1%). HIV‐TMA mortality is high despite TPE (49% of cases); better renal function reduces risk of TMA‐attributable death (HR 0.97, 95% CI 0.96–0.99, *p* < 0.001); use of FFP as infusant is associated with increased risk of mortality (HR 3.96, 95% CI 1.60–9.84, *p* = 0.003). Sepsis frequently complicates TPE (16.3% of courses) and contributes to excess mortality (20.7% of deaths); risk of infection increases with duration of TPE (OR 1.21, 95% CI 1.07–1.37, *p* = 0.002), implicating augmented immunosuppression in mortality. HIV infection parameters do not significantly affect risk of mortality or sepsis. Residual renal dysfunction is relatively rare in survivors at follow‐up.

**Conclusion:**

Mortality remains high in HIV‐TMA treated with TPE, and sepsis‐related complications are of concern. Randomized prospective studies are needed to evaluate the use of TPE versus plasma infusion (PI) and infusant choice in HIV‐TMA. Longer duration follow‐up studies are needed to evaluate residual renal dysfunction in survivors of HIV‐TMA.

## Summary

- HIV‐TMA is associated with advanced HIV infection.
- The disorder carries a significant risk of mortality despite treatment with therapeutic plasma exchange.
- Further prospective studies are needed to delineate the theoretical benefit of therapeutic plasma exchange over plasma infusion in the treatment of HIV‐TMA.

## 1 Introduction

The thrombotic microangiopathies (TMA) are a heterogenous group of rare disorders with an estimated annual incidence of 4 cases per million population in the developed world [1]. Data from the developing world is lacking; however, the South African National Blood Services (SANBS) recently estimated an annual incidence rate of 17.6–63.8 cases per million population through analysis of therapeutic plasma exchange (TPE) prescribing patterns [2].

This high incidence reflects the significant contribution of HIV as an aetiological factor in thrombotic microangiopathy (TMA) in the local context. Previous South African case series have reported that 77%–82% of patients diagnosed with TMA comprise people living with HIV (PLWH) [3, 4, 5, 6]. The importance of HIV as an aetiological factor in TMA is further evidenced by the disproportionately high representation of the virus as a cause of the disorder in case series from regions with lower seroprevalence rates [7].

Pathophysiological mechanisms underlying the development of HIV‐associated TMA (HIV‐TMA) have yet to be fully elucidated and may vary between patients. Endothelial dysfunction arising directly from cellular infection by the virus [8] or indirectly as a result of cellular injury from shed viral proteins and the chronic inflammatory milieu which accompanies HIV infection may precipitate the disorder in some patients [9]. In others, immune dysregulation associated with HIV infection may result in the development of autoantibodies to ADAMTS13 (*a d*isintegrin *a*nd *m*etalloproteinase with a *t*hrombo*s*pondin type 1 motif, type 13), an enzyme which cleaves ultra‐large von Willebrand factor (UL‐vWF) to exert an antithrombotic effect [10]. This variation in pathophysiological mechanisms in HIV‐TMA may account for lack of universally detectable ADAMTS13 deficiency or inhibitor levels in patients diagnosed with the disorder [10].

TMA is almost universally fatal without treatment; TPE reduces mortality to below 10% [11]. TPE has been reported to offer superior survival outcomes over plasma infusion (PI) as sole treatment [12]. This benefit of TPE is believed to derive from additive removal of UL‐vWF and ADAMTS13 inhibitor autoantibody, as well as ameliorating risk of fluid overload associated with high volume plasma infusion [13].

Mortality in HIV‐TMA has been reported to be higher than that in other forms of the disorder [14]. Prescription of TPE in the setting of the immunocompromised state of HIV infection may theoretically increase the risk of treatment‐related infections, contributing to this increased mortality [14, 15]. Assessment of the efficacy and safety of TPE in HIV‐TMA is limited by small patient numbers in case series [3, 4, 16, 17, 18], and complicated by restriction of TPE to a rescue therapy in patients failing first‐line therapy with plasma infusion (PI) [5, 19].

Long‐term sequelae of TMA remain poorly reported. Up to 45% of TMA survivors may manifest new‐onset hypertension, with 21% demonstrating chronic kidney disease (CKD) in the form of reduced estimated glomerular filtration rate (eGFR); 18% require ongoing dialytic support [20]. Although TMA has been suggested to be an important cause of acute kidney injury (AKI) in South Africa in PLWH [21], characterisation of long‐term renal outcomes in survivors of HIV‐TMA is lacking.

We have previously reported our experience in the presentation and outcome of TMA in 68 PLWH [6]. We here expand on this cohort to better describe the presentation and outcomes of HIV‐TMA, including a novel analysis of infection as a complication of TPE therapy, the role of plasma substitute in survival outcomes, and residual renal deficit in survivors at outpatient follow‐up.

## 2 Materials and Methods

A retrospective review of all patients receiving therapeutic plasma exchange (TPE) for treatment of presumed HIV‐associated thrombotic microangiopathy (HIV‐TMA) at our institution during the period 1 January 2010–31 December 2020 was undertaken. Permission to undertake this study was obtained from the University of the Witwatersrand Human Ethics Research Committee (protocol number M201166). In view of the retrospective nature of this work a waiver was provided for patient informed consent. The study was undertaken in accordance with the principles of the Declaration of Helsinki.

Patients treated for HIV‐TMA were retrospectively identified from the South African National Blood Services TPE database and through review of clinical records. The PLASMIC (*p*latelet count; combined haemo*l*ysis variable, absence of *a*ctive cancer; absence of *s*tem‐cell or solid organ transplant; *M*CV; *I*NR; and *c*reatinine) score [22] is not validated for TMA categories which do not feature ADAMTS13 deficiency [9] such as HIV‐TMA [10] and was therefore not used in this series. Diagnosis of HIV‐TMA was instead retrospectively confirmed by the presence of microangiopathic haemolysis (anaemia with peripheral blood schistocytes, and increased serum lactate dehydrogenase (LDH) activity above 190 IU/mL) and thrombocytopenia (platelet count below 140 × 109/mm^3^) with international normalised ratio (INR) below 1.5 and no other cause evident, consistent with previous case series [3, 5]. Pregnant women, patients with positive autoimmune serology, and those diagnosed with malignancy were excluded from analysis.

Standard treatment for HIV‐TMA during this series at this institution comprised daily TPE exchanging 1.5 plasma volumes with either thawed fresh frozen plasma (FFP) or cryo‐poor plasma, until remission. Remission is defined in our practice as improvement of LDH activity to below 200 IU/mL and platelet count to above 140 × 109/mm3 [23]. Hydrocortisone 50 mg intravenously daily 30 min before TPE was additionally prescribed with paracetamol 1000 mg *per os* and promethazine 25 mg intramuscular injection to reduce risk of anaphylactic reaction during treatment. Patients who do not show response to initial plasma substitute are converted at the discretion of the treating physician to TPE with the alternate substitute; thus, patients not responding to FFP are converted to TPE with cryo‐poor plasma. Augmented immunosuppression in our institution is reserved for those patients who fail to respond to TPE [23]. Caplacizumab, a monoclonal, bivalent humanised immunoglobulin fragment that binds to von Willebrand Factor to prevent platelet binding, which is recommended in both idiopathic and HIV‐associated forms of TMA [24], is not available in South Africa and was not prescribed to patients included in this cohort. All patients additionally received emergent initiation of antiretroviral therapy (ART).

Anonymised data was extracted from clinical records and stored in a secured database prior to export to Stata version 17 (StataCorp LLC, College Station, Texas, USA) for analysis. Demographics, HIV‐related disease status, and presentation of HIV‐TMA were characterised for the cohort. ADAMTS13 activity measurement is not routinely undertaken at our institution due to resource limitations and is not universally reduced in cases of HIV‐TMA [10]. We therefore did not include this parameter in this retrospective study. The Shapiro Wilk test was used to test the normality of distribution of included continuous variables. Central tendency of continuous variables was presented by the median and dispersion measures by the interquartile range; categorical variables were presented as number (n) and percentage (%). Presenting parameters were compared between plasma substitute groups using Kruskal Wallis ANOVA and Pearson Chi‐square testing for continuous variables and for categorical variables, respectively. All‐cause and TMA‐attributed inpatient mortality during TPE treatment was investigated using Cox proportional hazards modelling censored for termination of TPE upon disease remission, starting from the first daily session of TPE and using the number of daily TPE sessions to either inpatient death or disease remission as event times. Markers of HIV‐TMA severity at presentation (haemoglobin concentration, platelet count, renal function as determined by eGFR, and neurological deficit), as well as those evidencing or affecting severity of HIV infection (CD4 count, HIV viral load, and premorbid ART prescription), demographic data (age and gender), and plasma substitute (FFP or cryo‐poor plasma) were selected *a priori* as factors for analysis in this model. Patients receiving sequential FFP and cryo‐poor plasma were excluded from this analysis. Kaplan‐Meier survival curves were plotted for FFP and cryo‐poor plasma substitution groups and compared using the logrank test. Ascribed aetiologies of inpatient death were also characterised as were complications of TPE. Factors affecting the development of complications of treatment were modelled using logistic regression. Residual kidney dysfunction as determined by estimated glomerular filtration rate (eGFR) was determined for all survivors at discharge and for those survivors completing at least 3 months of outpatient follow‐up. In the latter group factors affecting renal function at follow‐up were modelled using linear regression analysis. A *p* < 0.05 was considered indicative of statistical significance.

## 3 Results

A total of 98 separate episodes of HIV‐associated thrombotic microangiopathy (HIV‐TMA) occurred in 94 patients during this study. Recurrences were documented in four patients. One patient suffered a relapse of HIV‐TMA during inpatient follow‐up and three patients presented with HIV‐TMA recurrence at 5, 16, and 31 months after initial presentation. In two of these cases recurrence was associated with ART default; one patient represented with HIV‐TMA whilst on ART treatment but with unsuppressed viral load (247 copies/mL). As shown in Table 1, presentation with HIV‐TMA was associated with other features of advanced HIV infection and was the index presentation of HIV infection in 53.3% of patients. Amongst 47 PLWH prior to diagnosis of HIV‐TMA, 26 (55.3% of patients known to be living with HIV) had defaulted ART, with 16 patients (34% of those known to be HIV positive) having been prescribed ART for more than 6 months prior to presentation. Reflecting this, only 6 (6.1%) of patients presented with a suppressed HIV viral load.

**Table 1:** Demographics and presentation of patients diagnosed with HIV‐TMA.

	*N* (%) or median (interquartile range)
Age (years)	35 (32–43)
Sex	
Female	66 (67.3%)
Male	32 (32.6%)
Ethnicity	
Black African	92 (93.9%)
Non‐black	6 (6.1%)
CD4 count (x10^6^/mm^3^)	151 (81–291)
Viral load (copies/mL)	117500 (922–728500)
Viral load suppression (< 20 copies/mL)	6 (6.1%)
On antiretroviral therapy	21 (21.4%)
On ART therapy longer than 6 months	16 (16.3%)
Defaulted ART prior to admission	26 (26.5%)
Newly diagnosed HIV positive	51 (52.0%)
Main indication for presentation	
Neurological deficit	
Confusion	53 (54.1%)
Focal neurology/seizures	6 (6.1%)
Headache	2 (2.0%)
Haemorrhagic manifestations	
Bleeding	19 (19.4%)
Anaemia symptoms	12 (12.2%)
Petechiae	3 (3.1%)
Other	
Non‐specific abdominal symptoms	2 (2.0%)
Fever	1 (1.0%)
Objective pyrexia	8 (8.2%)
Temperature (°C)	36.2 (36.1–36.3)
Haemoglobin (g/dL)	6.6 (5.5–8.2)
LDH (IU/mL)	1172 (788–1984)
Platelet count (x10^9^/mm^3^)	15 (12–21)
Creatinine (μmol/L)	109 (80–190)
eGFR (mL/min/1.73m^2^)	55 (29–73)

Neurological deficit was the leading reason for presentation, accounting for 61 patients (62.2%) seeking medical care; neurological deficit was additionally recorded in 12 patients presenting for other manifestations of TMA. Confusion was the most common form of neurological deficit (53 cases), followed by focal neurology (6 cases). Focal deficits comprised seizures (3 cases), hemiplegia (2 cases), and acute loss of consciousness (1 case). Haemorrhagic manifestations were the next most common indication for admission (34 cases, 34.7%). Active bleeding was the most frequent haemorrhagic complication (19 cases), comprising 8 cases of epistaxis, 4 cases of spontaneous bruising, 3 cases of gum bleeding, 2 cases each of haematemesis, and one case each of vaginal bleeding and melaena stool.

Forty‐nine cases (50%) were treated with TPE using thawed FFP as plasma substitute while 34 (34.7%) received TPE using cryo‐poor plasma. The remaining 15 cases received sequential prescription of thawed FFP and cryo‐poor plasma during TPE; in these cases, poor response to initial prescription of thawed FFP resulted in a change to use of cryo‐poor plasma. No significant differences in presenting parameters were detectable between plasma substitute groups (Table 2); although patients deemed requiring change of plasma substitute from FFP to cryo‐poor were more frequently female (*p* = 0.065). No patient in this series received augmented immunosuppression.

**Table 2:** Presenting parameters compared between plasma substitute groups.

	FFP (*N* = 49)	Cryo‐poor (*N* = 34)	Sequential FFP and cryo‐poor (*N* = 15)	*p*
Age (years)	35 (33–42)	38 (32–45)	34 (30–38)	0.411^a^
Female sex (*N*, %)	31 (63.3%)	21 (61.8%)	14 (93.3%)	0.065^b^
CD4 count (x 10^6^/mm^3^)	129 (83–251)	158 (69–285)	218 (83–471)	0.535^a^
HIV viral load (copies/mL)	120400 (1733–865000)	55100 (8530–408000)	392450 (224–1890000)	0.699^a^
Premorbid ART prescription (*N*, %)	8 (16.3%)	8 (23.5%)	5 (33.3%)	0.348^b^
Haemoglobin (g/dL)	6.6 (5.6–7.5)	6.4 (5.2–8.2)	7.2 (5.6–10)	0.509^a^
Platelet count (x10^9^/mm^3^)	15 (12–22)	14.5 (12–19)	18 (11–27)	0.962^a^
eGFR (mL/min/1.73m^2^)	46 (27–72)	58 (32–78)	59 (46–92)	0.235^a^
Neurological deficit (*N*, %)	34 (69.4%)	25 (73.5%)	14 (93.3%)	0.176^b^

^a^ Kruskall Wallis ANOVA test.

^b^ Pearson Chi square test.

Forty‐eight patients in this cohort demised despite receiving TPE therapy, with a crude inpatient mortality rate of 49%. Death was attributed to TMA in 34 cases (70.8% of mortalities; 34.7% of all treated cases). One additional patient demised from complications arising from intracerebral haemorrhage, itself attributable to thrombocytopaenia caused by HIV‐TMA. The remaining 13 mortalities (27.1%) were attributed to sepsis. The median number of TPE treatments received amongst mortalities was 2 (interquartile range 1–10 exchanges). The number of TPE treatments received by patients in whom mortality was attributed to HIV‐TMA was significantly lower than those in whom mortality was attributed to sepsis (median number of TPE exchanges 1 *vs.* 7, *p* < 0.001). Less severe renal dysfunction at presentation as evidenced by higher eGFR was associated with a reduction in both all‐cause inpatient mortality and inpatient mortality adjusted for deaths due to sepsis (Table 3).

**Table 3:** Factors affecting inpatient mortality during TPE treatment.

	HR	95% CI	*p*
**Unadjusted inpatient mortality**			
Age (years)	0.97	0.93–1.01	0.098
Female sex	1.02	0.49–2.15	0.952
CD4 count (x 10^6^/mm^3^)	0.99	0.99–1.00	0.270
HIV viral load (copies/mL)	1.00	1.00–1.00	0.114
Premorbid ART prescription	0.89	0.21–3.74	0.874
Haemoglobin (g/dL)	1.01	0.81–1.24	0.961
Platelet count (x 10^9^/mm^3^)	0.99	0.97–1.03	0.953
eGFR (mL/min/1.73m^2^)	0.97	0.96–0.98	< 0.001
Neurological deficit	1.53	0.63–3.72	0.3492
Use of FFP as substitute	2.72	1.31–5.63	0.007
**Inpatient mortality adjusted for deaths attributed to HIV‐TMA**			
Age (years)	0.96	0.92–1.00	0.079
Female sex	1.04	0.45–2.43	0.918
CD4 count (x 10^6^/mm^3^)	0.99	0.99–1.00	0.543
HIV viral load (copies/mL)	1.00	1.00–1.00	0.133
Premorbid ART prescription	1.36	0.31–6.07	0.683
Haemoglobin (g/dL)	0.94	0.74–1.20	0.631
Platelet count (x 10^9^/mm^3^)	0.99	0.96–1.02	0.553
eGFR (mL/min/1.73m^2^)	0.97	0.96–0.99	< 0.001
Neurological deficit	0.85	0.35–2.07	0.717
Use of FFP as substitute	3.96	1.60–9.84	0.003

*Note:* HR, hazard ratio; 95% CI, 95% confidence interval for hazard ratio.

**Figure 1:**
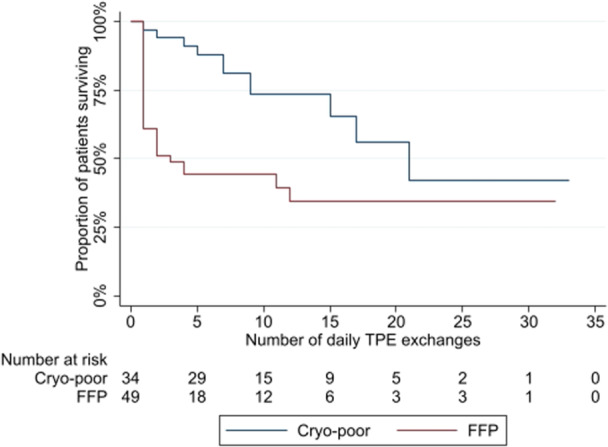
All‐Cause inpatient mortality by plasma infusant.

**Figure 2:**
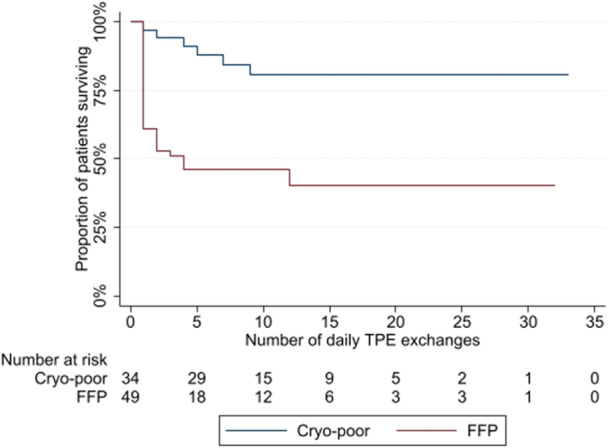
Mortality attributed to HIV‐TMA by plasma infusant.

Use of FFP compared to cryo‐poor plasma as plasma substitute was additionally strongly associated with risk of inpatient mortality. Poorer survival for patients receiving FFP was observed in terms of all‐cause mortality (logrank *p* = 0.003, Figure 1) and in terms of risk of death due to HIV‐TMA (logrank *p* < 0.001, Figure 2). Mortalities ascribed to HIV‐TMA were significantly more frequent (*p* = 0.002) in those receiving FFP as substitute (26 mortalities, 56.5% of patients receiving FFP) than in those receiving cryo‐poor (6 mortalities, 20.7% of patients receiving cryo‐poor).

Female sex (HR 0.22, 95% CI 0.05–0.97, *p* = 0.046) reduced risk of sepsis‐related inpatient mortality. A marginal effect was observed for higher CD4 count at presentation on sepsis‐related mortality risk reduction (HR 0.99, 95% CI 0.98–1.00, *p* = 0.043). No difference was observed in risk of death due to sepsis between plasma infusants (logrank *p* = 0.226).

A median of 10 TPE sessions were required to induce remission in survivors of HIV‐TMA (interquartile range 8–17). The number of exchanges required to induce remission was not dissimilar between patients receiving FFP as substitution plasma (median 9.5, interquartile range 5–17 exchanges) and those receiving cryo‐poor plasma (median 10, interquartile range 8–15 exchanges, *p* = 0.551). Survivors receiving sequential FFP and cryo‐poor plasma required a median of 14 exchanges (interquartile range 10–18 exchanges) to induce remission. CD4 count, HIV viral load, and premorbid prescription of ART therapy had no effect on the overall duration of TPE required to induce remission in survivors of HIV‐TMA (Table 4).

**Table 4:** Factors affecting duration of TPE in survivors of HIV‐TMA.

	ß	SE ß	*p*
Age (years)	0.22	0.18	0.247
Female sex	0.21	0.19	0.274
CD4 count (x 10^6^/mm^3^)	0.16	0.21	0.430
HIV viral load (copies/mL)	−0.13	0.20	0.521
Premorbid ART prescription	0.01	0.24	0.971
Haemoglobin (g/dL)	−0.03	0.19	0.521
Platelet count (x 10^9^mm^3^)	0.02	0.19	0.788
eGFR (mL/min/1.73m^2^)	−0.05	0.19	0.788
Use of FFP as substitute	−0.02	0.18	0.894

*Note:* ß, correlation co‐efficient; SE ß, standard error for correlation co‐efficient.

Complications of TPE therapy were reported in 40 treatment courses (40.8%). The most common complication of therapy was access dysfunction (26 cases, 26.5% of treatment courses), followed by infection/sepsis (16 cases, 16.3% of treatment courses). Citrate (2 cases) and anaphylactic reactions (1 case) were rarely encountered. The odds of developing complications of TPE therapy were increased by the duration of TPE therapy received but reduced in females (Table 5).

**Table 5:** Factors affecting the development of complications of TPE therapy.

	OR	95% CI	*p*
**Factors affecting risk of development of any complication**			
Age (years)	1.00	0.93–1.08	0.935
Female sex	0.14	0.03–0.63	0.011
Number of TPE treatments	1.42	1.19–1.71	< 0.001
CD4 count (x 10^6^/mm^3^)	0.99	0.99–1.00	0.546
HIV viral load (copies/mL)	1.00	0.99–1.00	0.700
Premorbid ART prescription	0.49	0.06–3.95	0.502
Use of FFP as substitute	3.28	0.73–14.81	0.121
**Factors affecting risk of development of infection or sepsis**			
Age (years)	1.02	0.94–1.09	0.658
Female sex	0.12	0.02–0.83	0.032
Number of TPE treatments	1.21	1.07–1.37	0.002
CD4 count (x 10^6^/mm^3^)	0.99	0.99–1.00	0.226
HIV viral load (copies/mL)	1.00	0.99–1.00	0.290
Premorbid ART prescription	1.02	0.10–10.01	0.989
Use of FFP as substitute	0.95	0.22–4.05	0.946

*Note:* OR, odds ratio; 95% CI, 95% confidence interval for odds ratio.

**Figure 3:**
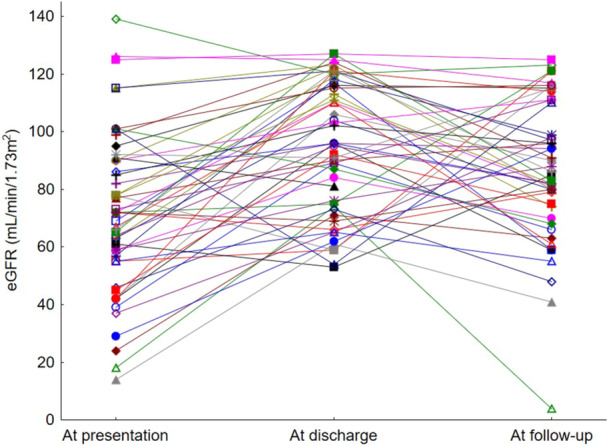
Renal function at follow‐up in survivors of TMA‐HIV.

Median eGFR at discharge in the 50 survivors of HIV‐TMA in this series was 96 mL/min/1.73m^2^ (interquartile range 74–117 mL/min/1.73m^2^). Thirty‐four survivors completed more than 3 months of outpatient follow‐up with a median duration of follow‐up of 49.7 months (Figure 3). The median eGFR in these patients was 87 mL/min/1.73m^2^ (interquartile range 75–110 mL/min/1.73m^2^).

Three of these patients (8.8%) met criteria for the presence of chronic kidney disease using a definition of eGFR below 60 mL/min/1.73m^2^ present for 3 or more months (Table 6). The development of inpatient complications of TPE therapy was associated with poorer renal function at outpatient follow‐up (Table 7).

**Table 6:** eGFR in Survivors with Long‐Term Follow‐Up.

	At presentation	At discharge	At follow‐up
	*N* (%)	*N* (%)	*N* (%)
eGFR ≥ 90 mL/min/1.73m^2^	8 (23.5%)	19 (55.9%)	16 (47.1%)
eGFR 60–90 mL/min/1.73m^2^	13 (38.2%)	11 (32.3%)	15 (44.1%)
eGFR 45–60 mL/min/1.73m^2^	3 (8.8%)	4 (11.8%)	1 (2.9%)
eGFR 30–45 mL/min/1.73m^2^	1 (2.9%)	0 (0%)	1 (2.9%)
eGFR 15–30 mL/min/1.73m^2^	4 (11.8%)	0 (0%)	0 (0%)
eGFR < 15 mL/min./1.73m^2^ or dialysis	5 (14.7%)	0 (0%)	1 (2.9%)

**Table 7:** Factors affecting renal function at outpatient follow‐up.

	ß	SE ß	*p*
CD 4 count (x 10^6^/mm^3^)	0.04	0.22	0.866
HIV viral load (copies/mL)	0.14	0.20	0.482
Number of TPE exchanges received	0.44	0.25	0.090
Any inpatient TPE complication	−0.60	0.27	0.036
Infection/sepsis TPE‐related complication	−0.29	0.22	0.198
FFP as plasma substitute	0.26	0.19	0.182
Time to outpatient eGFR measurement (days)	0.13	0.20	0.534

*Note:* ß, correlation co‐efficient; SE ß, standard error correlation co‐efficient.

## 4 Discussion

This large cohort confirms that presentation with TMA is associated with advanced HIV infection. Inpatient mortality in HIV‐associated TMA remains increased even with treatment with therapeutic plasma exchange; choice of infusant may affect risk of death due to HIV‐TMA. Infection‐related complications of treatment contribute to morbidity and mortality. Risk of developing an infectious complication is increased with longer duration of TPE therapy. CD4 count exerts a small but statistically significant additive risk of mortality due to sepsis in the setting of advanced immunocompromise. Residual renal dysfunction in survivors is mild and may reflect episodes of treatment‐related acute kidney injury.

Women and persons of Black African ethnicity are over‐represented in this study and in other TMA registry series. Gender disparities in the prevalence of autoimmune disease and the effects of oestrogen on endothelial function may underlie the female preponderance of patients presenting with the disorder [25, 26]. Low frequency of HLA‐DRB01*04 in Black African populations has been reported to increase the risk of TMA in these patients [27]. Historically entrenched gender and racial inequalities in South Africa place the greatest burden of HIV infection on young woman of Black African ethnicity [28]. The selection for a population at higher risk of TMA by the demographics of the HIV pandemic in the local context likely explains the high incidence of HIV‐TMA in South African studies [3, 4, 5, 6].

The clinical presentation of HIV‐TMA has been reported to feature more frequent neurological deficit [6, 19] and more severe haematological abnormality [6, 10, 18, 19] than forms of the disorder. It remains unclear whether these findings reflect TMA severity or the tendency for central nervous system involvement [29] and dyshaematopoeisis [9] by the HI virus. The association between HIV‐TMA and advanced stage of HIV infection shown in this and other studies [3, 4, 5, 6, 17, 18, 19] in combination with a more severe clinical presentation may be expected to translate to poorer survival outcomes in this disorder.

Early studies of the disorder suggested increased mortality risk in HIV‐TMA [30, 31]. Subsequent series have suggested improvement in survival outcomes [3, 17, 19], possibly reflecting the adoption of emergent ART prescription as a core component of therapy. More recent South African studies have reported similar survival outcomes for both PLWH and HIV negative patients [5, 6]. These latter reports may reflect the inclusion of a significant number of systemic lupus erythematosus (SLE) associated TMA cases in HIV negative patient cohorts [6]; a form of the disorder known to carry increased risk of mortality [32]. The crude inpatient mortality rate for HIV‐TMA in this series of 49% and that quoted by Masoet et al of 43.9% [5] are significantly higher than that reported for cases of immune‐mediated TMA in HIV negative patients of 5%–10% [11].

We found no evidence for any association between HIV viral load and CD4 count in mortality risk, which may be attributable to widespread advanced infection at presentation in our cohort. While some studies have shown an association between such parameters and treatment response [4, 17], others have found no such effect [3]. Analysis of our cohort suggests instead a weak but discernible effect for reduced mortality risk with better preserved renal function as evidenced by higher eGFR at presentation. This is consistent with data previously reported in immune‐mediated TMA in HIV negative patients [33]. Uraemia is unlikely to explain this association, since significant renal dysfunction was unusual in this series. Instead, more severe renal dysfunction may serve as a marker for the involvement of other target organs; in particular, the risk of myocardial infarction due to coronary thrombotic microangiopathy [34].

The present study includes to our knowledge the first analysis of the effect of plasma substitute type on survival outcomes in HIV‐TMA. Substantial improvement in survival in patients receiving cryo‐poor plasma in this series is of interest given the overall high mortality rate reported. The apparent benefit of cryo‐poor plasma in reducing TMA‐related mortality has been suggested in other series [35, 36], and is thought to derive from lower UL‐vWF levels present in this plasma substitute [35]. Low UL‐vWF content in cryo‐poor plasma limits platelet adhesion and thrombotic events which contribute to mortality [36]. The quantitative reduction in UL‐vWF capacity for platelet adhesion with cryo‐poor plasma has an analogous effect to the blockade of platelet adhesion to UL‐vWF engendered by caplacizumab [36]. Given the prohibitive cost [37] and resultant lack of access to caplacizumab, cryo‐poor plasma may represent an attractive therapeutic option in economically disadvantaged regions with high incidence rates of HIV‐TMA such as South Africa.

Infection complicated 16% of treatment courses and sepsis contributed more than a quarter of mortalities in this series. Data on infection risk in patients receiving TPE is sparce. An infection rate of 0.02% per treatment course has previously been reported in a large series of patients undergoing TPE for a variety of indications [38]. Rates of up to 48% of treatment courses have been reported in critically ill patients receiving TPE in intensive care settings [39]. Kinetic models suggest that sequential reductions in antibody levels during TPE result in 90% of antibody being removed after 5 exchanges [40]. Increased risk of sepsis‐related mortality with increased number of TPE performed is therefore probable evidence of the cumulative effect of TPE in augmenting the immunocompromise of advanced HIV infection in this series.

Use of TPE in HIV‐TMA at our institution follows treatment guidelines [23, 24] and is predicated on the theoretical benefit of UL‐vWF removal and higher volume of plasma substitute infusion [13]. Previous retrospective South African series have reported favourable outcomes with plasma infusion (PI) alone [5, 19]. The median duration of TPE required to induce remission in survivors of HIV‐TMA in our cohort (10 days) is identical to that reported by Masoet et al using PI as sole therapy; the mortality rate in the current series (49%) may evidence an increased risk of mortality compared to that reported by those authors (43.9%) [5]. Inpatient mortality attributable to HIV‐TMA is similar in these cohorts (34% and 32%, respectively) [5], suggesting the contribution of sepsis‐related deaths to the excess mortality in the present study, substantiating concerns regarding TPE‐augmented immunocompromise in PLWH.

Together, these observations suggest that risk of precipitating infection through use of TPE in HIV‐TMA may outweigh the reported benefits of this therapeutic strategy in other forms of the disorder. Instead, PI using cryo‐poor plasma when caplacizumab is unavailable may constitute a lower risk, more cost‐effective treatment protocol. Confirmation of this hypothesis will require future prospective randomized control trials.

We were unable to discern any predictor for duration of TPE required to induce remission in survivors of HIV‐TMA. Sequential prescription of plasma substitute may evidence more refractory cases of HIV‐TMA not otherwise retrospectively detectable. Of note, patients requiring sequential plasma substitute had more frequent neurological deficit (93.3% compared to 69.4% for those receiving only FFP and 73.5% for those receiving only cryo‐poor; *p* = 0.176) and were more often women (93.3% compared to 63.3% and 61.8%, respectively). Neurological deficit has been reported to convey an adverse prognosis in other HIV‐TMA series [4]; gender‐based differences in endothelial dysfunction [26] may account for apparent refractoriness in women, if present. Previous reports have suggested an increased duration of TPE in patients with HI viral loads in excess of 500 000 copies/mL [17], although subsequent series have failed to demonstrate an association between HIV infection parameters and treatment duration [3]. Such differences between studies may evidence different pathophysiological processes underlying HIV‐TMA and warrant further investigation.

Residual kidney dysfunction is an under‐reported outcome in most TMA series. Numerically significant improvement in renal function from admission was observed at attainment of remission in this cohort, which persisted at long‐term follow‐up. Significant renal dysfunction was rare in this cohort, with a median eGFR at follow‐up of 87 mL/min.1,73m^2^ and only 8.7% of survivors manifesting an eGFR below 60 mL/min/1.73m^2^. The apparent association between inpatient treatment complication and eGFR at follow‐up may suggest a role for preventable acute kidney injury episodes on long‐term kidney dysfunction in survivors. Longer duration of outpatient follow‐up is needed to better categorise the long‐term effects of HIV‐TMA mediated AKI on risk of chronic kidney disease in survivors.

There are limitations to this study. The retrospective nature of this work is likely to have compromised accurate recording of clinical data such as the nature and severity of neurological deficit, which may in turn affect analysis of the contribution of such parameters to mortality outcomes [4]. We also acknowledge the lack of ADAMTS13 data in this cohort occasioned by resource constraints. Although not required for the diagnosis of HIV‐TMA, ADAMTS‐13 activity may delineate mortality risk and would be a useful adjunct to analysis of this outcome [7]. The retrospective nature of this study additionally requires reliance on contemporaneous assessment of the causes of mortality which may have compromised accurate description of this outcome. Disparate duration of outpatient follow‐up, caused by patient default after discharge, additionally limits full evaluation of long‐term renal outcomes. The use of eGFR as sole indicator of CKD without consideration of the presence of proteinuria, occasioned by limited availability of the latter measurement due to the retrospective nature of this study, further limits full assessment of residual renal dysfunction in this cohort. Finally, the single‐centre nature of this study may raise concerns regarding the generalizability of our findings. However, we would suggest that this latter limitation conversely provides for homogeneity in the case definition and management of HIV‐TMA, thus limiting confounding factors.

## 5 Conclusions

HIV‐associated thrombotic microangiopathy occurs in the setting of advanced HIV infection. Poorer survival in HIV‐TMA with therapeutic plasma exchange compared to that reported for other forms of the disorder may partially be explained by exacerbation of HIV‐related immunocompromise by this therapy as evidenced by significant sepsis‐related complications. Comparison with previous studies of plasma infusion as sole therapy does not appear to show superiority of TPE in HIV‐TMA. Further evaluation through prospective randomized trials is needed to better evaluate the efficacy of TPE against PI and to determine the optimal plasma infusant in HIV‐TMA. Longer duration of follow‐up in survivors is additionally required to better categorise renal sequelae.

## Author Contributions

**Malcolm Davies:** conceptualization, methodology, data curation, formal analysis, supervision, project administration, writing – original draft, writing – review and editing. **Sheetal Chiba:** writing – original draft, writing – review and editing. **Sayuri Harishun:** conceptualization, methodology, data curation, investigation, project administration. **Chandni Dayal:** writing – review and editing. **Zaheera Cassimjee:** conceptualization, methodology, writing – original draft, writing – review and editing.

## Funding

The authors received no specific funding for this work.

## Ethics Statement

Approval to undertake this study was provided by the Human Research Ethics Committee of the University of the Witwatersrand, Johannesburg (protocol number M201166). In view of the retrospective nature of this study a waiver was provided for patient informed consent. The study was undertaken in accordance with the principles of the Declaration of Helsinki.

## Conflicts of Interest

The authors declare no conflicts of interest.

## Transparency Statement

The lead author Malcolm Davies affirms that this manuscript is an honest, accurate, and transparent account of the study being reported; that no important aspects of the study have been omitted; and that any discrepancies from the study as planned (and, if relevant, registered) have been explained.

## Data Availability

Data are available in a public, open access repository accessible at https://doi.org/10.54223/20.500.12430/551197.
